# 2-[(4-Bromo­phen­yl)sulfan­yl]-2-meth­oxy-1-phenyl­ethan-1-one: crystal structure, Hirshfeld surface analysis and computational chemistry

**DOI:** 10.1107/S2056989019006765

**Published:** 2019-05-17

**Authors:** Ignez Caracelli, Julio Zukerman-Schpector, Henrique J. Traesel, Paulo R. Olivato, Mukesh M. Jotani, Edward R. T. Tiekink

**Affiliations:** a Departamento de Física, Universidade Federal de São Carlos, 13565-905 São Carlos, SP, Brazil; b Departamento de Química, Universidade Federal de São Carlos, 13565-905 São Carlos, SP, Brazil; c Instituto de Química, Universidade de São Paulo, 05508-000 São Paulo, SP, Brazil; d Department of Physics, Bhavan’s Sheth R. A. College of Science, Ahmedabad, Gujarat 380001, India; e Research Centre for Crystalline Materials, School of Science and Technology, Sunway University, 47500 Bandar Sunway, Selangor Darul Ehsan, Malaysia

**Keywords:** crystal structure, sulfan­yl, phenyl­ethanone, Hirshfeld surface analysis, NCI plots, computational chemistry

## Abstract

The title compound, C_15_H_13_BrO_2_S, comprises three different substituents bound to a central (and chiral) methine-C atom, *i.e*. (4-bromo­phen­yl)sulfanyl, benzaldehyde and meth­oxy residues: crystal symmetry generates a racemic mixture. A twist in the mol­ecule is evident about the methine-C—C(carbon­yl) bond as evidenced by the O—C—C—O torsion angle of −20.8 (7)°. The dihedral angle between the bromo­benzene and phenyl rings is 43.2 (2)°, with the former disposed to lie over the oxygen atoms. The most prominent feature of the packing is the formation of helical supra­molecular chains as a result of methyl- and methine-C—H⋯O(carbon­yl) inter­actions. The chains assemble into a three-dimensional architecture without directional inter­actions between them. The nature of the weak points of contacts has been probed by a combination of Hirshfeld surface analysis, non-covalent inter­action plots and inter­action energy calculations. These point to the importance of weaker H⋯H and C—H⋯C inter­actions in the consolidation of the structure.

## Abstract 2

The title mol­ecule is twisted about the methine-C—C(carbon­yl) bond [the O—C—C—O torsion angle is −20.8 (7)°] and the dihedral angle between the bromo­benzene and phenyl rings is 43.2 (2)°.

## Chemical context   

Recently, the crystal structure determination of the chloro analogue of the title compound was described (Caracelli *et al.*, 2018 ▸). This was evaluated as a part of on-going studies into the conformational and electronic characteristics of various β-thio­carbonyl, β-bis-thio­carbonyl and β-thio-β-oxacarbonyl compounds, and their selenium counterparts, employing infrared spectroscopy, computational chemistry and X-ray crystallographic methods (Vinhato *et al.*, 2013 ▸; Zukerman-Schpector *et al.*, 2015 ▸; Caracelli *et al.*, 2015 ▸; Traesel *et al.*, 2018 ▸). In particular, the evaluation of the anti-inflammatory activity of what could be selective COX-2 inhibitors (Cerqueira *et al.*, 2017 ▸) motivates these investigations, which are supported by mol­ecular docking studies designed to ascertain the mechanism(s) of inhibition (Baptistini, 2015 ▸). Subsequently, crystals of the title bromo analogue (I) were obtained: the crystal structure is reported herein along with an analysis of the calculated Hirshfeld surfaces, non-covalent inter­action plots (for selected inter­actions) as well as a computational chemistry study.
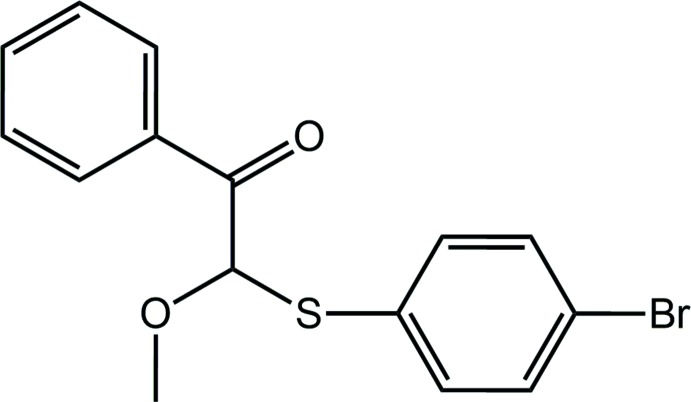


## Structural commentary   

**Figure 1:**
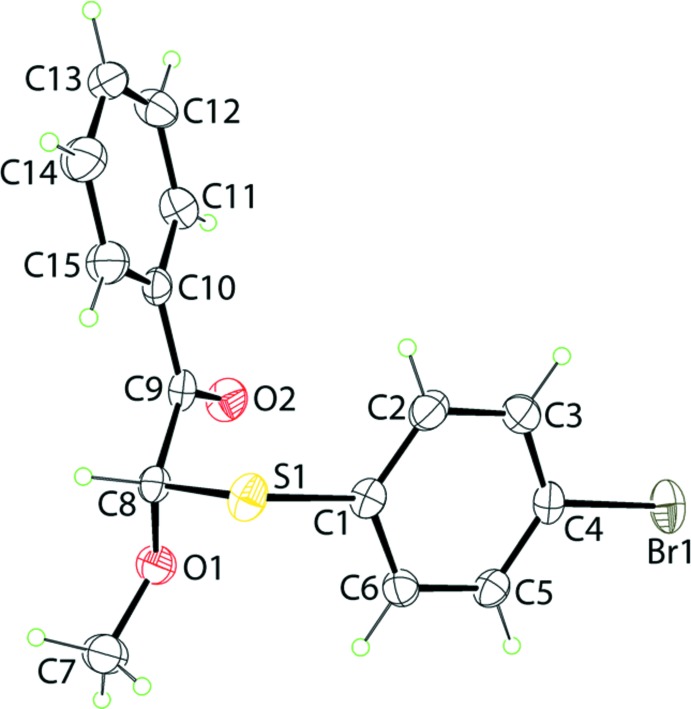
The mol­ecular structure of (I), showing the atom-labelling scheme and displacement ellipsoids at the 25% probability level.

The mol­ecular structure of (I), Fig. 1 ▸, is isostructural with the previously described chloro analogue, (II) (Caracelli *et al.*, 2018 ▸). Here, the central chiral methine-C8 atom is connected to (4-bromo­phen­yl)sulfanyl, phenyl­ethanone and meth­oxy groups. There is a twist in the ethanone residue as seen in the value of the O1—C8—C9—O2 torsion angle of −20.8 (7)°, with the oxygen atoms being approximately *syn*. The dihedral angle between the bromo­benzene and phenyl rings is 43.2 (2)°, indicative of an inclined relative disposition. Globally, the bromo­benzene ring is orientated towards the ethanone residue.

**Figure 2:**
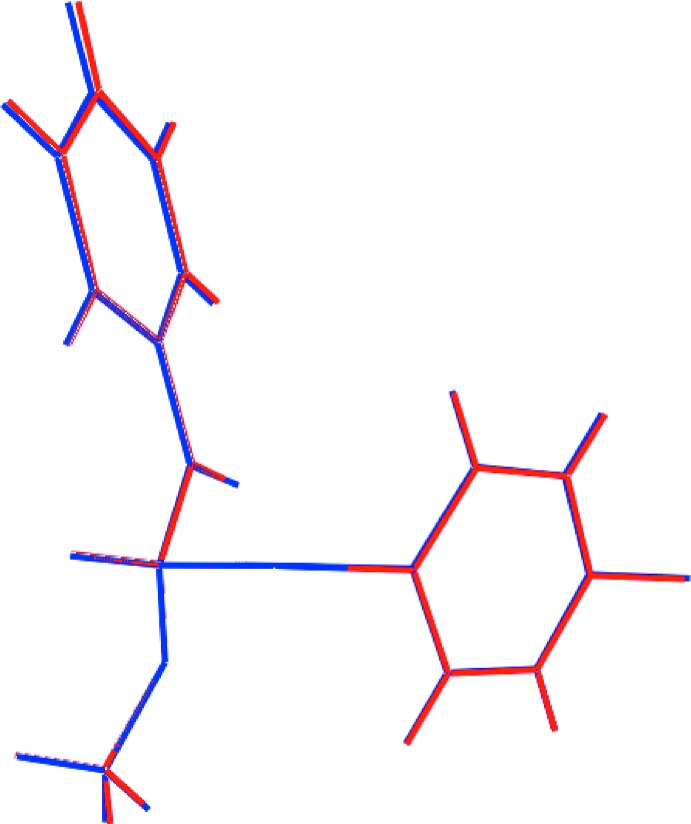
Overlay diagram of (I) (red image) and (II) (blue image).

The geometric parameters in (I) can be compared with those of (II): the twist about the central C8—C9 bond is approximately the same in (II), *i.e*. the the O1—C8—C9—O2 torsion angle is 19.3 (7)°, as is the dihedral angle of 42.9 (2)° between the aromatic rings. The overlay diagram in Fig. 2 ▸ highlights the close similarity between the mol­ecular structures of (I) and (II).

## Supra­molecular features   

**Figure 3:**
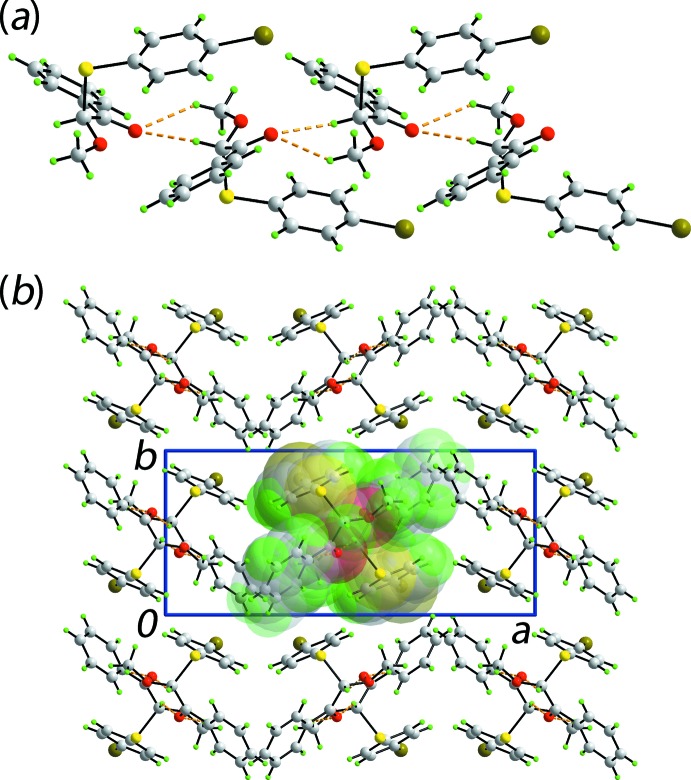
Mol­ecular packing in (I): (*a*) view of the helical supra­molecular chain parallel to the *c* axis sustained by C—H⋯O inter­actions shown as orange dashed lines and (*b*) view of the unit-cell contents shown in projection down the *c* axis; one chain is highlighted in space-filling mode.

The main feature of the mol­ecular packing of (I) is the presence of C—H⋯O inter­actions where the carbonyl-O2 atom accepts two contacts from methyl-C7-H and methine-C8-H atoms derived from the same mol­ecule to generate six-membered {⋯O⋯HCOCH} synthons, Table 1 ▸. The result is a supra­molecular chain propagating along [001] with an helical topology (2_1_ symmetry), Fig. 3 ▸(*a*). The chains pack without directional inter­actions between them, Fig. 3 ▸(*b*).

## Hirshfeld surface analysis   

The Hirshfeld surface calculations for (I) were performed in accord with protocols described recently (Tan *et al.*, 2019 ▸) employing *Crystal Explorer* (Turner *et al.*, 2017 ▸). Over and above the analysis of the important surface contacts in the crystal of (I), the results are compared with those for the recently determined isostructural chloro analogue (II) (Caracelli *et al.*, 2018 ▸). The crystal of (I) has similar inter­molecular C—H⋯O inter­actions (Table 1 ▸) and short inter­atomic H⋯H, C⋯H and C⋯C contacts (Table 2 ▸) as in isostructural (II), as detailed below.

**Figure 4:**
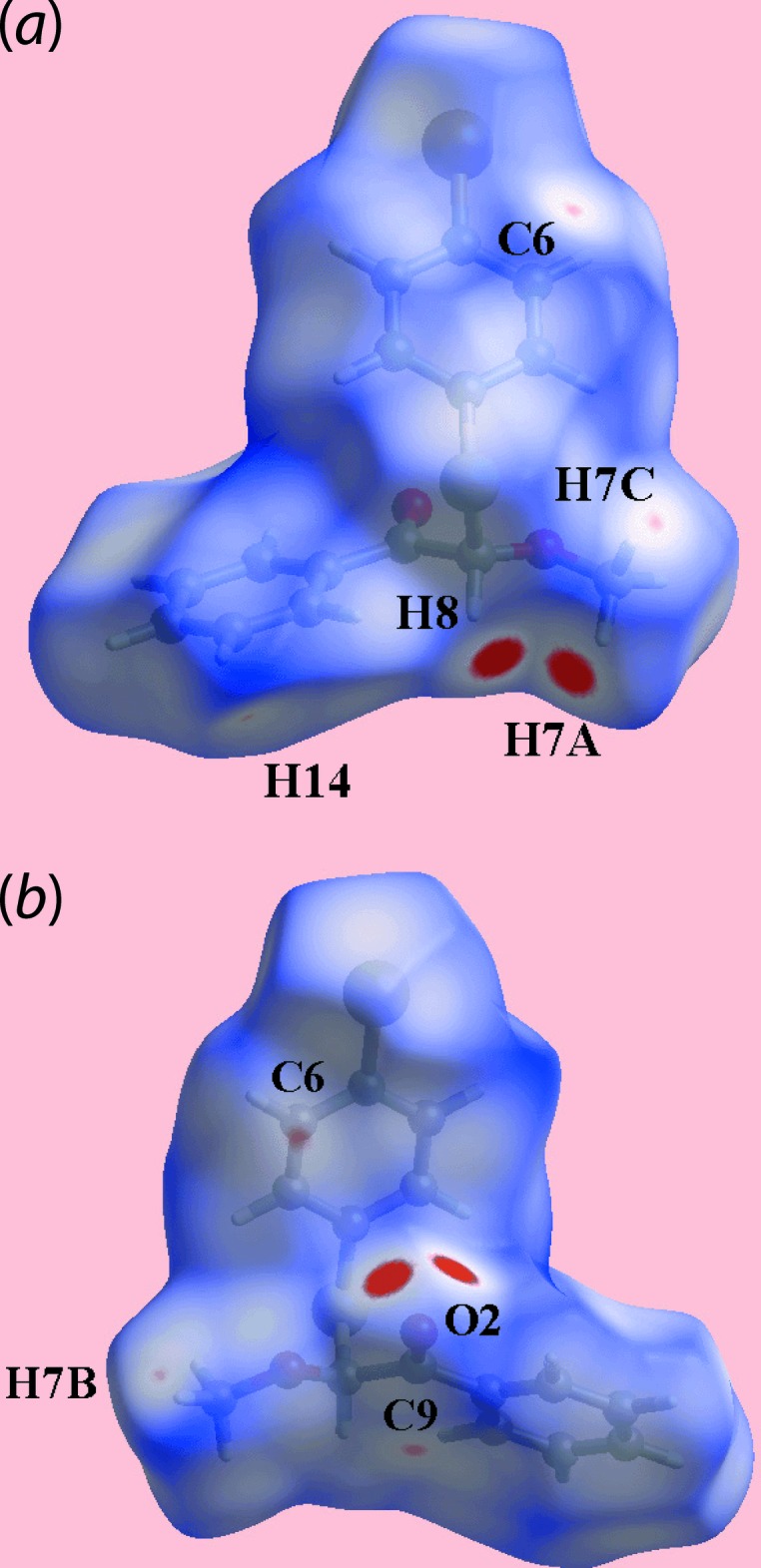
Two views of the Hirshfeld surface for (I) mapped over *d*_norm_ in the range −0.084 to +1.422 arbitrary units.

**Figure 5:**
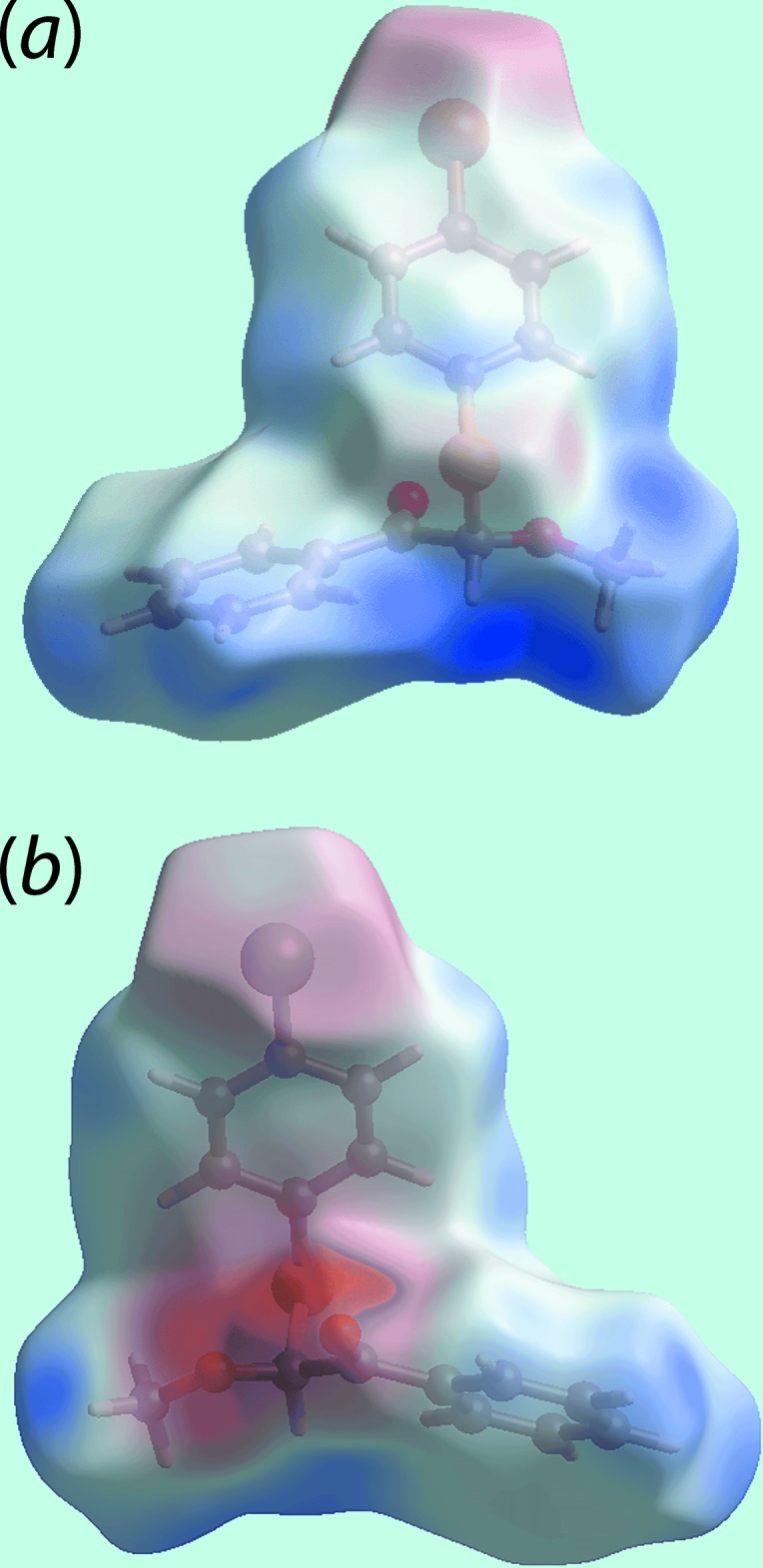
Two views of the Hirshfeld surface for (I) mapped over the electrostatic potential in the range −0.074 to + 0.053 atomic units. The red and blue regions represent negative and positive electrostatic potentials, respectively.

**Figure 6:**
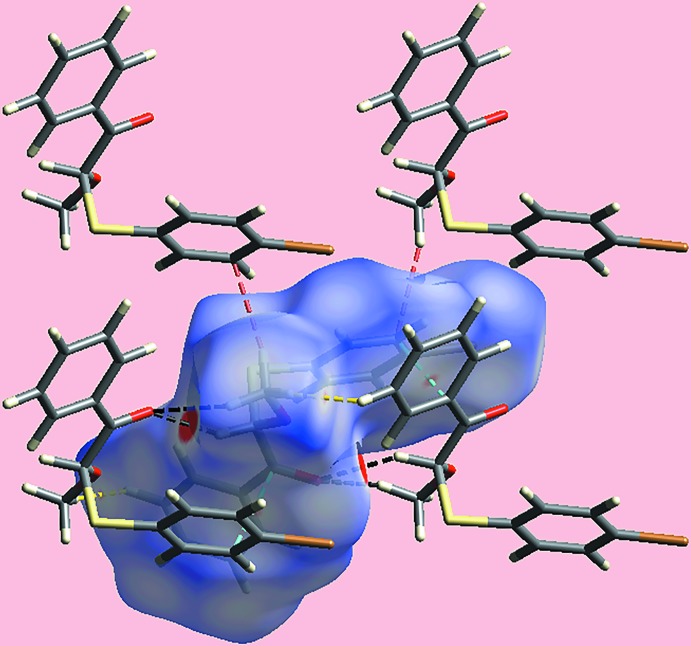
A view of the Hirshfeld surface for (I) mapped over *d*_norm_ in the range −0.084 to +1.422 arbitrary units highlighting inter­molecular C—H⋯O, C⋯C, H⋯H and C⋯H/H⋯C contacts by black, red, yellow and sky-blue dashed lines, respectively.

The inter­molecular contacts in (I), Tables 1 ▸ and 2 ▸, are characterized as the pair of bright-red spots near the carbonyl-O2 atom, and each of the methyl-H7*A* and methine-H8 atoms on the Hirshfeld surfaces mapped over *d*_norm_ in the images of Fig. 4 ▸. Further, inter­actions are indicated by the faint-red spots near the methyl-H7*B* and H7*C*, phenyl-H14, bromo­benzene-C6 and carbonyl-C9 atoms in Fig. 4 ▸. On the Hirshfeld surfaces mapped over the calculated electrostatic potential in the images of Fig. 5 ▸, the donors and acceptors of inter­molecular inter­actions are viewed as blue and red regions around the participating atoms corresponding to positive and negative potentials, respectively. The environment around a reference mol­ecule within the *d*_norm_-mapped Hirshfeld surface highlighting the inter­molecular C—H⋯O inter­actions and short inter­atomic H⋯H, C⋯H/H⋯C and C⋯C contacts is illus­trated in Fig. 6 ▸.

**Figure 7:**
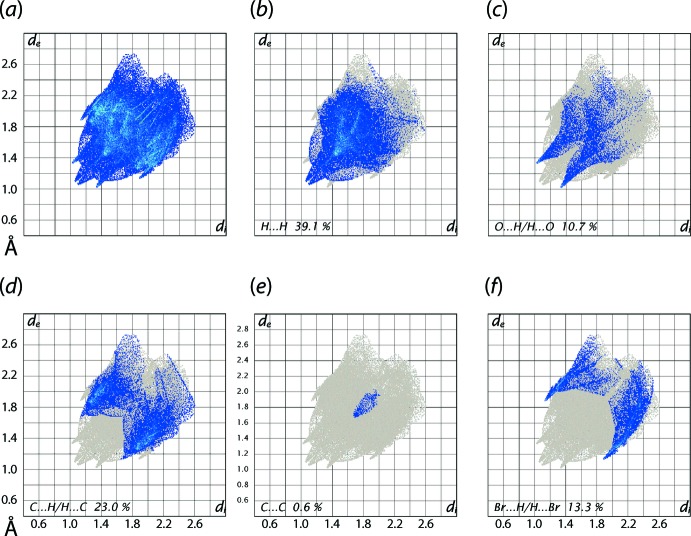
(*a*) The full two-dimensional fingerprint plot for (I) and (*b*)–(*f*) those delineated into H⋯H, O⋯H/H⋯O, C⋯H/H⋯C, C⋯C and Br⋯H/H⋯Br contacts.

From the overall two-dimensional fingerprint plot in Fig. 7 ▸(*a*), and also those delineated into H⋯H, O⋯H/H⋯O, C⋯H/H⋯C, C⋯C and Br⋯H/H⋯Br contacts in Fig. 7 ▸(*b*)–(*f*), respectively, it is evident that the plots are basically identical in shape to those calculated for the chloro analogue (II) with only slight differences in the distribution of points (Caracelli *et al.*, 2018 ▸). The percentage contributions from the different inter­atomic contacts to the Hirshfeld surfaces of (I) and (II) are summarized in Table 3 ▸; these values again highlight the similarities between (I) and (II).

The C—H⋯O contacts significant in the crystal of (I), Table 1 ▸, are represented as the pair of spikes at *d*_e_ + *d*_i_ ∼2.3 Å in the fingerprint plot delineated into O⋯H/H⋯O contacts, Fig. 7 ▸(*c*). The short inter­atomic H⋯H, C⋯H/H⋯C and C⋯C contacts (Table 2 ▸) are characterized as pair of beak-shape tips at *d*_e_ + *d*_i_ ∼2.1 Å, Fig. 7 ▸(*b*), and forceps at *d*_e_ + *d*_i_ ∼2.8 Å, Fig. 7 ▸(*d*), and vase-shaped distribution of points at *d*_e_ + *d*_i_ ∼3.3 Å, Fig. 7 ▸(*e*), in the respective delineated fingerprint plots. In addition to these contacts, the crystal also features short inter­atomic Br⋯H/H⋯Br contacts appearing as the pair of forceps-like tips at *d*_e_ + *d*_i_ ∼3.0 Å in the delineated fingerprint plot of Fig. 7 ▸(*f*). The small contribution from other remaining inter­atomic contacts summarized in Table 3 ▸ have a negligible effect on the packing.

## Inter­action energies   

The pairwise inter­action energies between the mol­ecules within the crystal are calculated by the summation of four energy components comprising electrostatic (*E*_ele_), polarization (*E*_pol_), dispersion (*E*_dis_) and exchange-repulsion (*E*_rep_) (Turner *et al.*, 2017 ▸). These energies were obtained by using the wave function calculated at the HF/STO-3G level theory for each of (I) and (II). The individual energy components as well as total inter­action energy relative to reference mol­ecule within the mol­ecular cluster were calculated. Table 4 ▸ summarizes qu­anti­tatively the strength and nature of inter­molecular inter­actions in the crystals of (I) and (II).

It is observed from the inter­action energies calculated between the reference mol­ecule and the symmetry-related mol­ecules at *R* = 6.40 and 6.13 Å (where *R* is the separation of the centres of gravity of the mol­ecules), respectively (Table 4 ▸), that the almost identical values of the electrostatic energy component are due to inter­molecular C—H⋯O inter­actions whereas the dispersive components are dominant owing to the short inter­atomic contacts between the same mol­ecules. The other short inter­atomic C⋯H/H⋯C contact between the methyl-H7*C* and phenyl-C6 atoms in (I) and (II), and the H12⋯Br1 contact in (I) have a major contribution from dispersion components.

**Figure 8:**
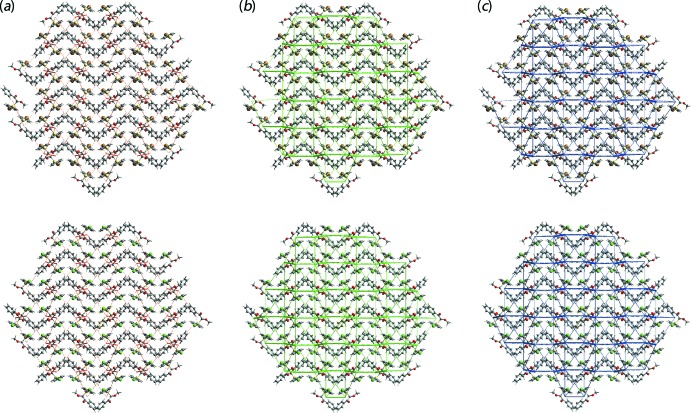
A comparison of the energy frameworks, plotted with the same scale, composed of (*a*) electrostatic potential force, (*b*) dispersion force and (*c*) total energy for the mol­ecules of (I), upper images, and (II), lower images, all viewed down the *c*-axis direction. same scale factor of 50 with a cut-off value of 3 kJ mol^−1^ within 4 *x* 4 *x* 4 unit cells.

The magnitudes of inter­molecular energies are represented graphically in the energy frameworks for (I) and (II) viewed down the *c* axes are shown in Fig. 8 ▸. Here, the supra­molecular architecture of the crystals is represented as cylinders joining centroids of mol­ecular pairs. The red, green and blue coloration represent the energy components *E*_ele_, *E*_disp_ and *E*_tot_, respectively. The radius of the cylinder is proportional to the magnitude of inter­action energy which are adjusted to the same scale factor (3 kJ mol^−1^) within 4 × 4 × 4 unit cells. From the energy frameworks for (I) and (II) illustrated in Fig. 8 ▸, it is clearly evident that the supra­molecular associations viewed down the *c* axis are identical, reflecting the isostructural relationship between (I) and (II).

## Non-covalent inter­action plots   

**Figure 9:**
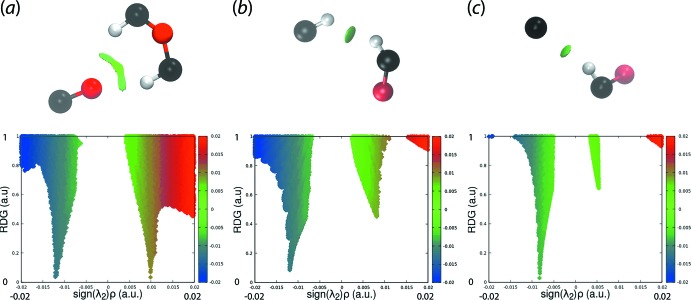
Non-covalent inter­action plots for inter­molecular inter­actions between (*a*) each of the methyl-C7- and methine-C—H atoms and the carbonyl-O2 atom, (*b*) the methyl-H7*B* and phenyl-H14 atoms and (*c*) bromo­benzene-C6 and methyl-H7*C* atoms.

The non-covalent inter­action plot (NCIplot) analysis was used in the present study in order to confirm the attractive nature of some of the specified inter­molecular contacts (Contreras-García *et al.*, 2011 ▸). This method is based on the electron density and its derivatives allowing the visualization of the gradient isosurfaces. The colour-based isosurfaces correspond to the values of sign(λ^2^)*ρ*(r), where *ρ* is the electron density and λ^2^ is the second eigenvalue of the Hessian matrix of *ρ* (Johnson *et al.*, 2010 ▸). The isosurfaces for the inter­actions between the carbonyl-O2 and each of the methyl-H7*B* and phenyl-H14 atoms, the H7*B* and H14 atoms, and the chloro­benzene-C6 and methyl-H7*C* atoms are shown in the upper views of Fig. 9 ▸(*a*)–(*c*), respectively. The green isosurface observed in each of these indicates a weakly attractive inter­action as opposed to attractive (blue isosurface) or repulsive (red). The lower views of Fig. 9 ▸, where the plots of the RDG versus sign(λ^2^)*ρ*(r) are depicted, the non-covalent inter­action peaks appear at density values equal or lower than 0.01 a.u., consistent with weakly attractive inter­actions.

## Database survey   

There are three literature structures related to (I), namely the already mentioned (II) (NIBTAW; Caracelli *et al.*, 2018 ▸), the S-bound 4-meth­oxy­benzene derivative [(III); JUPLOZ; Caracelli *et al.*, 2015 ▸] and the S-bound 4-tolyl species [NOVGIQ; (IV); Zukerman-Schpector *et al.*, 2015 ▸] derivatives. All four compounds crystallize in the ortho­rhom­bic space group *Pca*2_1_ and are isostructural. The differences between the mol­ecules of (I)–(IV) relates to the relative orientations of the S-bound meth­oxy­benzene ring in (III). This comes about owing to a twist about the C8—S1 bond as manifested in the C4—S1—C8—C9 torsion angles of 57.1 (4), 57.3 (5), 46.6 (3) and 57.9 (3)° for (I)–(IV), respectively. This difference notwithstanding, the angles between the S-bound benzene rings and the phenyl rings in (I)–(IV) span a relatively narrow range of values, *i.e*. 43.2 (2), 42.9 (2), 40.11 (16) and 44.03 (16)°, respectively.

## Synthesis and crystallization   

Firstly, 4′-bromo­thio­phenol (10.0 g, 52.9 mmol) was reacted with bromine (3.1 ml, 56.0 mmol) in di­chloro­methane (400 ml) on a hydrated silica gel support (50 g of SiO_2_ and water (30 ml) to give 4′-bromo­phenyl di­sulfide (8.0 g, yield 80%). A brown solid was obtained after filtration and evaporation without further purification (Ali & McDermott, 2002 ▸). Then, a solution of 2-meth­oxy aceto­phenone (Sigma–Aldrich; 1.0 ml, 7.3 mmol) in THF (25 ml), was added dropwise to a cooled (195 K) solution of diiso­propyl­amine (1.1 ml, 8.0 mmol) and *n*-butyl­lithium (5.4 ml, 7.3 mmol) in THF (30 ml). After 30 mins, a solution of 4′-bromo­phenyl di­sulfide (2.8 g, 7.3 mmol) with hexa­methyl­phospho­ramide (HMPA) (1.3 ml, *ca* 7.3 mmol) dissolved in THF (35 ml) was added dropwise to the enolate solution (Zoretic & Soja, 1976 ▸). After stirring for 3 h, water (70 ml) was added at room temperature and extraction with diethyl ether ensued. The organic layer was then treated with a saturated solution of ammonium chloride until neutral pH was reached and then dried over anhydrous magnesium sulfate. A brown oil was obtained after evaporation of the solvent. Purification through flash chromatography with *n*-hexane was used in order to remove the non-polar reactant (di­sulfide), then with dry acetone to give a mixture of both aceto­phenones (product and reactant). Crystallization was performed by vapour diffusion of *n*-hexane into a chloro­form solution held at 283 K to give the pure product (0.6 g, yield = 70%). Irregular colourless crystals suitable for X-ray diffraction of (I) were obtained by the same pathway. M.p. 357.0–357.5 K. ^1^H NMR (CDCl_3_, 500 MHz, δ ppm): 3.67 (*s*, 3H), 5.87 (*s*, 1H), 7.20–7.23 (*m*, 2H), 7.39–7.41 (*m*, 2H), 7.44–7.47 (*m*, 2H), 7.57–7.62 (*m*, 1H), 7.92–7.94 (*m*, 2H). ^13^C NMR (CDCl_3_, 125 MHz, δ p.p.m.): 190.16, 135.73, 134.18, 133.53, 132.13, 129.92, 128.81, 128.57, 123.41, 89.28, 56.10. Microanalysis calculated for C_15_H_13_BrO_2_S (%): C 53.42, H 3.89. Found (%): C 53.19, H 3.85. High-Resolution MS [*M* ^+^, *M^2^* ^+^] calculated: 335.9820, 337.9799; found: 335.9797, 337.9778.

## Refinement details   

Crystal data, data collection and structure refinement details are summarized in Table 5 ▸. The carbon-bound H atoms were placed in calculated positions (C—H = 0.93–0.98 Å) and were included in the refinement in the riding-model approximation, with *U*_iso_(H) set to 1.2–1.5*U*_eq_(C). The absolute structure was determined based on differences in Friedel pairs included in the data set (Parsons *et al.*, 2013 ▸).

## Supplementary Material

Crystal structure: contains datablock(s) I, global. DOI: 10.1107/S2056989019006765/hb7825sup1.cif

Structure factors: contains datablock(s) I. DOI: 10.1107/S2056989019006765/hb7825Isup2.hkl

Supporting information file. DOI: 10.1107/S2056989019006765/hb7825Isup3.cml

CCDC reference: 1915470

Additional supporting information:  crystallographic information; 3D view; checkCIF report

## Figures and Tables

**Table 1:** Hydrogen-bond geometry (Å, °)

*D*—H⋯*A*	*D*—H	H⋯*A*	*D*⋯*A*	*D*—H⋯*A*
C7—H7*A*⋯O2^i^	0.96	2.47	3.296 (9)	144
C8—H8⋯O2^i^	0.98	2.44	3.331 (6)	150

Symmetry code: (i) 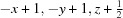.

**Table 2:** Summary of short inter­atomic contacts (Å) in (I) and (II)

Contact	Distance	Symmetry operation
	(I)	
H7*B*⋯H14	2.15	1 − *x*, − *y*, − 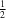 + *z*
H7*C*⋯C6	2.74	1 − *x*, 2 − *y*, 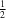 + *z*
H12⋯Br1	3.02	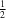 − *x*, −1 + *y*, 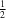 + *z*
C6⋯C9	3.355 (8)	1 − *x*, 1 − *y*, − 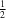 + *z*
		
	(II)	
H7*B*⋯H14	2.10	1 − *x*, − *y*, 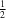 + *z*
H7*B*⋯C14	2.76	1 − *x*, − *y*, 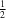 + *z*
H7*C*⋯C6	2.73	1 − *x*, 1 − *y*, 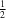 + *z*
C6⋯C9	3.334 (9)	1 − *x*, − *y*, 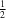 + *z*

Notes: (*a*) The inter­atomic distances are calculated in *Crystal Explorer* (Turner *et al.*, 2017 ▸) whereby the *X*—H bond lengths are adjusted to their neutron values.

**Table 3:** Percentage contributions of inter­atomic contacts to the Hirshfeld surface for (I) and (II)

Contact	Percentage contribution	
	(I), *X* = Br	(II), *X* = Cl
H⋯H	39.3	39.1
O⋯H/H⋯O	11.0	10.7
C⋯H/H⋯C	23.2	23.0
*X*⋯H/H⋯*X*	12.8	13.3
S⋯H/H⋯S	4.4	4.3
*X*⋯S/S⋯*X*	2.1	2.3
*X*⋯O/O⋯*X*	2.1	2.1
C⋯O/O⋯C	1.5	1.5
C⋯*X*/*X*⋯C	1.5	1.8
C⋯S/S⋯C	1.2	1.1
C⋯C	0.6	0.6

**Table 4:** Summary of inter­action energies (kJ mol^−1^) calculated for (I) and (II)

Contact	*R* (Å)	*E*_ele_	*E*_pol_	*E*_dis_	*E*_rep_	*E*_tot_
(I)						
C7—H7*A*⋯O2^i^ +						
C8—H8⋯O2^i^ +						
H7*B*⋯H14^i^ +						
C6⋯C9^i^	6.40	−20.0	−12.1	−53.2	34.0	−48.0
H7*C*⋯C6^ii^	8.75	−7.0	−1.2	−16.7	9.3	−15.4
H12⋯Br1^ii^	10.83	−4.1	−0.9	−12.9	6.4	−11.2
(II)						
C7—H7*A*⋯O2^iii^ +						
C8—H8⋯O2^iii^ +						
H7*B*⋯H14^iii^ +						
C6⋯C9^iii^ +						
H7*B*⋯C14^iii^	6.13	−19.5	−11.8	−52.7	35.1	−46.6
H7*C*⋯C6^iv^	9.06	−6.6	−1.4	−14.5	8.2	−14.0

Notes: Symmetry operations: (i) 1 − *x*, 1 − *y*, −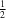 + *z*; (ii) 1 − *x*, 2 − *y*, 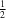 + *z*; (iii) 1 − *x*, − *y*, 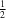 + *z*; (iv) 1 − *x*, 1 − *y*, 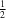 + *z*.

**Table 5:** Experimental details

Crystal data	
Chemical formula	C_15_H_13_BrO_2_S
*M*_r_	337.21
Crystal system, space group	Orthorhombic, *P* *c* *a*2_1_
Temperature (K)	293
*a*, *b*, *c* (Å)	18.0683 (13), 8.0190 (6), 9.8513 (5)
*V* (Å^3^)	1427.35 (16)
*Z*	4
Radiation type	Mo *K*α
μ (mm^−1^)	3.02
Crystal size (mm)	0.47 × 0.20 × 0.14

Data collection	
Diffractometer	Bruker APEXII CCD
Absorption correction	Multi-scan (*SADABS*; Sheldrick, 1996 ▸)
*T*_min_, *T*_max_	0.545, 0.745
No. of measured, independent and observed [*I* > 2σ(*I*)] reflections	6329, 2820, 1903
*R*_int_	0.037
(sin θ/λ)_max_ (Å^−1^)	0.625

Refinement	
*R*[*F* ^2^ > 2σ(*F* ^2^)], *wR*(*F* ^2^), *S*	0.035, 0.086, 0.90
No. of reflections	2820
No. of parameters	173
No. of restraints	1
H-atom treatment	H-atom parameters constrained
Δρ_max_, Δρ_min_ (e Å^−3^)	0.24, −0.36
Absolute structure	Flack *x* determined using 702 quotients [(*I* ^+^)−(*I* ^−^)]/[(*I* ^+^)+(*I* ^−^)] (Parsons *et al.*, 2013 ▸)
Absolute structure parameter	0.013 (11)

Computer programs: *APEX2* and *SAINT* (Bruker, 2009 ▸), *SIR2014* (Burla *et al.*, 2015 ▸), *SHELXL2014* (Sheldrick, 2015 ▸), *ORTEP-3 for Windows* (Farrugia, 2012 ▸), *DIAMOND* (Brandenburg, 2006 ▸), *MarvinSketch* (ChemAxon, 2010 ▸) and *publCIF* (Westrip, 2010 ▸).

## supplementary crystallographic information

### Crystal data

**Table:**

C_15_H_13_BrO_2_S	*D*_x_ = 1.569 Mg m^−^^3^
*M**_r_* = 337.21	Mo *K*α radiation, λ = 0.71073 Å
Orthorhombic, *P**c**a*2_1_	Cell parameters from 1496 reflections
*a* = 18.0683 (13) Å	θ = 2.8–23.5°
*b* = 8.0190 (6) Å	µ = 3.02 mm^−^^1^
*c* = 9.8513 (5) Å	*T* = 293 K
*V* = 1427.35 (16) Å^3^	Irregular, colourless
*Z* = 4	0.47 × 0.20 × 0.14 mm
*F*(000) = 680	

### Data collection

**Table:**

Bruker APEXII CCD diffractometer	1903 reflections with *I* > 2σ(*I*)
φ and ω scans	*R*_int_ = 0.037
Absorption correction: multi-scan (SADABS; Sheldrick, 1996)	θ_max_ = 26.4°, θ_min_ = 2.3°
*T*_min_ = 0.545, *T*_max_ = 0.745	*h* = −22→22
6329 measured reflections	*k* = −10→7
2820 independent reflections	*l* = −10→12

### Refinement

**Table:**

Refinement on *F*^2^	Secondary atom site location: difference Fourier map
Least-squares matrix: full	Hydrogen site location: inferred from neighbouring sites
*R*[*F*^2^ > 2σ(*F*^2^)] = 0.035	H-atom parameters constrained
*wR*(*F*^2^) = 0.086	*w* = 1/[σ^2^(*F*_o_^2^)] where *P* = (*F*_o_^2^ + 2*F*_c_^2^)/3
*S* = 0.90	(Δ/σ)_max_ < 0.001
2820 reflections	Δρ_max_ = 0.24 e Å^−^^3^
173 parameters	Δρ_min_ = −0.36 e Å^−^^3^
1 restraint	Absolute structure: Flack *x* determined using 702 quotients [(*I*^+^)-(*I*^-^)]/[(*I*^+^)+(*I*^-^)] (Parsons *et al*., 2013)
Primary atom site location: structure-invariant direct methods	Absolute structure parameter: 0.013 (11)

### Special details

**Table:**

Geometry. All esds (except the esd in the dihedral angle between two l.s. planes) are estimated using the full covariance matrix. The cell esds are taken into account individually in the estimation of esds in distances, angles and torsion angles; correlations between esds in cell parameters are only used when they are defined by crystal symmetry. An approximate (isotropic) treatment of cell esds is used for estimating esds involving l.s. planes.

### Fractional atomic coordinates and isotropic or equivalent isotropic displacement parameters (Å^2^)

**Table:**

	*x*	*y*	*z*	*U*_iso_*/*U*_eq_	
C1	0.3809 (4)	0.7980 (8)	0.2687 (6)	0.0481 (16)	
C2	0.3282 (4)	0.7230 (8)	0.3471 (6)	0.0547 (17)	
H2	0.2844	0.6846	0.3086	0.066*	
C3	0.3411 (4)	0.7049 (8)	0.4862 (6)	0.0573 (18)	
H3	0.3059	0.6531	0.5406	0.069*	
C4	0.4057 (3)	0.7634 (8)	0.5432 (5)	0.0442 (14)	
C5	0.4582 (4)	0.8394 (7)	0.4612 (6)	0.0481 (15)	
H5	0.5020	0.8793	0.4989	0.058*	
C6	0.4456 (4)	0.8561 (8)	0.3225 (5)	0.0470 (15)	
H6	0.4808	0.9063	0.2671	0.056*	
C7	0.6000 (4)	0.6921 (10)	0.7438 (8)	0.074 (2)	
H7A	0.6041	0.6513	0.8351	0.111*	
H7C	0.5822	0.8049	0.7453	0.111*	
H7B	0.6477	0.6889	0.7009	0.111*	
C8	0.4826 (3)	0.5677 (7)	0.7324 (5)	0.0446 (14)	
H8	0.4910	0.5417	0.8284	0.053*	
C9	0.4446 (3)	0.4212 (7)	0.6661 (5)	0.0417 (14)	
C10	0.3832 (3)	0.3328 (7)	0.7355 (6)	0.0424 (13)	
C11	0.3535 (3)	0.1926 (8)	0.6732 (7)	0.0547 (17)	
H11	0.3720	0.1580	0.5897	0.066*	
C12	0.2968 (4)	0.1035 (8)	0.7336 (7)	0.0660 (18)	
H12	0.2773	0.0102	0.6904	0.079*	
C13	0.2689 (4)	0.1533 (9)	0.8592 (7)	0.067 (2)	
H13	0.2308	0.0938	0.9005	0.080*	
C14	0.2986 (4)	0.2922 (9)	0.9210 (7)	0.069 (2)	
H14	0.2800	0.3273	1.0043	0.083*	
C15	0.3551 (4)	0.3791 (10)	0.8614 (7)	0.0613 (18)	
H15	0.3751	0.4708	0.9060	0.074*	
O1	0.5500 (2)	0.5910 (6)	0.6703 (4)	0.0573 (11)	
O2	0.4633 (2)	0.3773 (6)	0.5525 (4)	0.0623 (12)	
S1	0.41992 (10)	0.7500 (2)	0.72103 (15)	0.0551 (4)	
Br1	0.36371 (4)	0.82410 (9)	0.07901 (8)	0.0718 (3)	

### Atomic displacement parameters (Å^2^)

**Table:**

	*U*^11^	*U*^22^	*U*^33^	*U*^12^	*U*^13^	*U*^23^
C1	0.060 (4)	0.045 (4)	0.039 (3)	0.011 (3)	−0.008 (3)	0.001 (3)
C2	0.046 (4)	0.060 (5)	0.059 (4)	0.001 (3)	−0.001 (3)	0.005 (3)
C3	0.062 (4)	0.054 (5)	0.056 (4)	−0.002 (4)	0.005 (3)	0.012 (3)
C4	0.050 (3)	0.044 (4)	0.039 (3)	0.007 (3)	0.006 (3)	0.000 (2)
C5	0.048 (4)	0.045 (4)	0.052 (3)	0.001 (3)	−0.006 (3)	0.000 (3)
C6	0.048 (4)	0.047 (4)	0.045 (3)	0.005 (3)	0.009 (3)	0.005 (2)
C7	0.073 (5)	0.091 (6)	0.058 (5)	−0.028 (4)	−0.002 (4)	0.001 (4)
C8	0.051 (4)	0.050 (4)	0.032 (2)	−0.004 (3)	−0.002 (3)	0.005 (3)
C9	0.054 (4)	0.041 (4)	0.030 (3)	0.012 (3)	−0.005 (3)	0.003 (2)
C10	0.049 (3)	0.038 (3)	0.041 (3)	0.005 (3)	−0.010 (3)	0.005 (3)
C11	0.055 (4)	0.055 (4)	0.055 (4)	0.006 (3)	−0.005 (3)	−0.008 (3)
C12	0.055 (4)	0.069 (5)	0.074 (4)	−0.012 (4)	−0.012 (4)	−0.003 (4)
C13	0.052 (5)	0.071 (5)	0.077 (5)	−0.009 (3)	−0.004 (4)	0.028 (4)
C14	0.074 (5)	0.079 (6)	0.056 (4)	−0.010 (4)	0.005 (4)	0.002 (4)
C15	0.071 (5)	0.072 (5)	0.042 (3)	−0.012 (4)	0.003 (3)	−0.004 (3)
O1	0.058 (3)	0.070 (3)	0.044 (2)	−0.009 (2)	0.003 (2)	−0.001 (2)
O2	0.081 (3)	0.068 (3)	0.037 (2)	−0.004 (2)	0.007 (2)	−0.009 (2)
S1	0.0774 (11)	0.0497 (9)	0.0382 (7)	0.0083 (9)	0.0041 (9)	−0.0035 (7)
Br1	0.0797 (5)	0.0905 (5)	0.0450 (3)	0.0229 (4)	−0.0087 (4)	0.0030 (4)

### Geometric parameters (Å, º)

**Table:**

C1—C6	1.365 (9)	C8—C9	1.510 (7)
C1—C2	1.366 (9)	C8—S1	1.853 (6)
C1—Br1	1.905 (6)	C8—H8	0.9800
C2—C3	1.398 (8)	C9—O2	1.221 (6)
C2—H2	0.9300	C9—C10	1.482 (8)
C3—C4	1.378 (9)	C10—C11	1.389 (8)
C3—H3	0.9300	C10—C15	1.391 (9)
C4—C5	1.388 (8)	C11—C12	1.384 (9)
C4—S1	1.773 (5)	C11—H11	0.9300
C5—C6	1.392 (8)	C12—C13	1.395 (10)
C5—H5	0.9300	C12—H12	0.9300
C6—H6	0.9300	C13—C14	1.378 (9)
C7—O1	1.414 (8)	C13—H13	0.9300
C7—H7A	0.9600	C14—C15	1.369 (10)
C7—H7C	0.9600	C14—H14	0.9300
C7—H7B	0.9600	C15—H15	0.9300
C8—O1	1.375 (6)		
			
C6—C1—C2	121.8 (5)	O1—C8—H8	108.7
C6—C1—Br1	118.9 (5)	C9—C8—H8	108.7
C2—C1—Br1	119.3 (5)	S1—C8—H8	108.7
C1—C2—C3	118.9 (7)	O2—C9—C10	119.5 (5)
C1—C2—H2	120.5	O2—C9—C8	119.6 (5)
C3—C2—H2	120.5	C10—C9—C8	120.8 (5)
C4—C3—C2	120.4 (6)	C11—C10—C15	118.0 (6)
C4—C3—H3	119.8	C11—C10—C9	118.1 (5)
C2—C3—H3	119.8	C15—C10—C9	123.8 (5)
C3—C4—C5	119.4 (5)	C12—C11—C10	120.9 (6)
C3—C4—S1	120.3 (5)	C12—C11—H11	119.5
C5—C4—S1	120.2 (5)	C10—C11—H11	119.5
C4—C5—C6	120.1 (6)	C11—C12—C13	120.1 (6)
C4—C5—H5	120.0	C11—C12—H12	119.9
C6—C5—H5	120.0	C13—C12—H12	119.9
C1—C6—C5	119.3 (6)	C14—C13—C12	118.9 (6)
C1—C6—H6	120.4	C14—C13—H13	120.6
C5—C6—H6	120.4	C12—C13—H13	120.6
O1—C7—H7A	109.5	C15—C14—C13	120.8 (7)
O1—C7—H7C	109.5	C15—C14—H14	119.6
H7A—C7—H7C	109.5	C13—C14—H14	119.6
O1—C7—H7B	109.5	C14—C15—C10	121.3 (7)
H7A—C7—H7B	109.5	C14—C15—H15	119.4
H7C—C7—H7B	109.5	C10—C15—H15	119.4
O1—C8—C9	108.4 (4)	C8—O1—C7	114.6 (5)
O1—C8—S1	114.0 (4)	C4—S1—C8	101.3 (3)
C9—C8—S1	108.1 (4)		
			
C6—C1—C2—C3	−0.2 (10)	O2—C9—C10—C15	−176.3 (6)
Br1—C1—C2—C3	−179.8 (5)	C8—C9—C10—C15	1.9 (8)
C1—C2—C3—C4	0.6 (10)	C15—C10—C11—C12	1.2 (9)
C2—C3—C4—C5	−0.5 (9)	C9—C10—C11—C12	179.2 (5)
C2—C3—C4—S1	176.7 (5)	C10—C11—C12—C13	−0.4 (9)
C3—C4—C5—C6	−0.1 (9)	C11—C12—C13—C14	0.2 (10)
S1—C4—C5—C6	−177.2 (5)	C12—C13—C14—C15	−0.7 (11)
C2—C1—C6—C5	−0.3 (9)	C13—C14—C15—C10	1.6 (11)
Br1—C1—C6—C5	179.3 (4)	C11—C10—C15—C14	−1.7 (10)
C4—C5—C6—C1	0.5 (9)	C9—C10—C15—C14	−179.6 (6)
O1—C8—C9—O2	−20.8 (7)	C9—C8—O1—C7	−163.5 (5)
S1—C8—C9—O2	103.2 (5)	S1—C8—O1—C7	76.1 (6)
O1—C8—C9—C10	160.9 (4)	C3—C4—S1—C8	102.0 (5)
S1—C8—C9—C10	−75.0 (5)	C5—C4—S1—C8	−80.9 (5)
O2—C9—C10—C11	5.7 (8)	O1—C8—S1—C4	63.5 (4)
C8—C9—C10—C11	−176.0 (5)	C9—C8—S1—C4	−57.1 (4)

### Hydrogen-bond geometry (Å, º)

**Table:**

*D*—H···*A*	*D*—H	H···*A*	*D*···*A*	*D*—H···*A*
C7—H7*A*···O2^i^	0.96	2.47	3.296 (9)	144
C8—H8···O2^i^	0.98	2.44	3.331 (6)	150

Symmetry code: (i) −*x*+1, −*y*+1, *z*+1/2.
